# Handgrip Evaluation Before and After Pulmonary Rehabilitation Therapy in Patients With Chronic Obstructive Pulmonary Disease (COPD)

**DOI:** 10.7759/cureus.69404

**Published:** 2024-09-14

**Authors:** Nestor A Diaz Posada, Diana J Cano Rosales, Maria C Amaya Muñoz, Mario A Buitrago Gomez, Silvia J Villabona, Paul Anthony Camacho López

**Affiliations:** 1 Internal Medicine, Fundación Oftalmológica de Santander, Bucaramanga, COL; 2 Pulmonology, Instituto Neumologico del Oriente, Bucaramanga, COL; 3 Research, Development, and Technological Innovation, Fundación Oftalmológica de Santander, Bucaramanga, COL; 4 Research, Development, and Technological Innovation, Fundación Oftalmológica de Santander, Floridablanca, COL

**Keywords:** chronic obstructive, hand strength, lung disease, pulmonary disease, quality of life, rehabilitation

## Abstract

Introduction

Chronic Obstructive Pulmonary Disease (COPD) is a systemic disease characterized by skeletal muscle dysfunction, leading to increased morbidity and mortality and deteriorating quality of life. Pulmonary rehabilitation therapy improves symptoms and long-term adherence. This study aimed to evaluate how COPD patients respond to pulmonary rehabilitation therapy and its correlation with handgrip strength measurements.

Materials and methods

A prospective cohort study was conducted in patients over 45 years old with a spirometric diagnosis of COPD from a specialized reference center in Bucaramanga. Handgrip strength was measured before and after completing the pulmonary rehabilitation program. Patients with neurological or cognitive impairments, decompensated cardiovascular disease, nutritional diseases, or those in a telerehabilitation program were excluded.

Results

Seventy-one patients were included in the study, with 66.2% completing follow-up after the program. The average age was 73.38 years (SD ±7.77), 53.52% were women, and 60.56% had a history of smoking. After the follow-up, the average handgrip strength delta was 1.64 kg (SD ±3.48) p<0.001, 74.47% of them representing a positive result after pulmonary rehabilitation program. A higher Charlson index correlated with a positive delta, while a negative delta correlated with a lower Charlson index (p=0.01). A positive handgrip strength delta was associated with higher baseline quality of life scores.

Conclusions

Periodic handgrip strength measurements predict frailty and muscle dysfunction in COPD patients. Pulmonary rehabilitation therapy is a simple and cost-effective intervention that correlates with the improvement of indirect prognosis and survival indicators.

## Introduction

Chronic Obstructive Pulmonary Disease (COPD) is considered by the World Health Organization as the fourth leading cause of mortality from non-communicable chronic diseases, with an estimated prevalence in the adult population in the United States of 6% for men and 1-3% for women [1]. It is a systemic disease with pulmonary and extrapulmonary manifestations. There is a correlation in skeletal muscle dysfunction resulting from decreased physical activity tolerance, systemic inflammation, hypoxemia, malnutrition, oxidative stress, and the use of systemic corticosteroids [2,3], leading to sedentarism, social isolation, depression, and physical deconditioning. This, combined with COPD exacerbations, results in a deterioration of quality of life [4,5].

From a pathophysiological perspective, there is a deterioration in the functionality of peripheral "non-respiratory" muscles due to systemic inflammatory factors causing an imbalance in which catabolism predominates, leading to muscle tissue loss, increased protein degradation, and minimal response to nutritional interventions, resulting in progressive muscle weakness [6]. This musculoskeletal system involvement advances as the disease progresses, initially losing lean mass and eventually altering body weight and body mass index (BMI) [7].

The dysfunction of peripheral muscles observed in COPD patients is the result of a multitude of pathophysiological changes occurring in skeletal muscles, which exhibit reduced oxidative capacity that can lead to early lactic acidosis [8], decreased muscle fiber volume [9], and abnormal capillarization of muscle fibers [10].

To estimate muscle involvement, various diagnostic tests have been studied, among which bioimpedance has shown a good correlation with clinical, functional, and effort variables in COPD patients, regardless of their stage [11].

Handgrip strength (HGS) provides an objective measure of the integrity and strength of the intrinsic hand muscles and forearm muscles. The most accurate and reproducible method is physical measurement with a dynamometer [12]. Therefore, HGS through handgrip measurement is proposed as a quick, simple, and economical method that provides useful information as a nutritional marker and predictor of morbidity and mortality in various populations [13], potentially serving as a quality of life marker to stratify risk in COPD patients [14].

HGS serves as a metric to assess muscle function and overall physical capacity. Particularly relevant to the aging population, HGS is significantly connected to the concept of sarcopenia, which encompasses the age-related decline in muscle mass, strength, and function. Evaluating HGS in clinical practice allows for the identification of patients at risk of muscle weakness, facilitates the early diagnosis of conditions such as sarcopenia, and monitors the response to therapeutic interventions. Furthermore, its easy implementation through portable, non-invasive devices makes it a valuable tool for routine assessment in various clinical settings, from outpatient consultations to hospital care [15].

Pulmonary rehabilitation (PR) is defined as a comprehensive intervention focusing on the individual patient, including exercise training, education, and behavior change, which improves long-term adherence to health-improving behaviors and increases tolerance to higher workloads [16,17]. Evidence supports PR as an intervention that alleviates dyspnea and fatigue [18] and reduces anxiety and depression [19,20].

Although HGS was evaluated in Colombia and Bucaramanga through the PURE study, and cut-off points for the population were defined [21], its relationship and impact on chronic respiratory diseases have not been evaluated. Therefore, the objective of this study was to evaluate the effect of PR therapy on HGS in COPD patients in a reference center in Bucaramanga.

## Materials and methods

Study design

We developed a prospective cohort study with all patients who met the inclusion criteria of the pulmonology center. Once the patient was initially evaluated, handgrip strength measurements and a 6MWT were conducted, followed by a six-month pulmonary rehabilitation program that included psychological and nutritional assessments, along with 48 pulmonary therapy sessions. The rehabilitation program consists of 48 sessions, held two to three times per week, each lasting one hour. The process begins with a nursing assessment where laboratory tests, nutritional and psychological evaluations, and spirometry are requested. After this, the patient enters the pulmonary rehabilitation program under the supervision of a rehabilitation specialist, who prescribes the appropriate exercise and adjusts the treatment during each session. Nursing, nutrition, and psychology departments conduct two consultations, one at the beginning and another at the end of the program. If the patient experiences an exacerbation during the process, an immediate consultation with a pulmonologist is scheduled. Upon completion of the program, participants were re-evaluated by a pulmonology specialist, and new handgrip strength and 6-minute walk test measurements were conducted.

Inclusion of participants

All patients over 45 years old with a spirometric diagnosis of COPD according to the GOLD 2020 guidelines who completed the PR program in a Pneumology Institute during the data collection period were included in the study, and convenience sampling was performed. Patients with cognitive or neurological impairments preventing understanding of interviewer instructions or execution of requested maneuvers, as well as those with decompensated cardiovascular disease, nutritional diseases, or in telerehabilitation programs, were excluded.

Data collection

Data was collected through interviews, medical record reviews, phone calls, and handgrip measurements before and after completing the PR therapy.

Handgrip measurement was obtained using a calibrated Camry hand dynamometer for HGS measurement of 200 pounds (Camry Industries, Kowloon, Hong Kong). The measurement was taken in a standing position with shoulders abducted, elbows flexed at 90°, and forearms in a neutral position. Maximum strength in both extremities was evaluated. Patients were instructed to squeeze the handle as hard as possible for 3-5 seconds. Three test repetitions were performed, with at least a 1-minute pause between repetitions.

Statistical analysis

In the univariate analysis, numerical variables were described using measures of central tendency and dispersion, while categorical variables were described using proportions. For the bivariate analysis, graphical and numerical methods were used to evaluate the normality of numerical variables, and since this assumption was not met, Mann-Whitney U and Kruskal-Wallis tests and Spearman correlation coefficient were used to assess the association between baseline HGS and its change between measurements and other variables of interest. To evaluate the change in HGS magnitude before and after PR, the delta of the second measurement with respect to the first was estimated, and the medians of HGS measurements before and after PR were compared using the Wilcoxon test. A significance level of 0.05 was established for all analyses, which were conducted using Stata 13.

Ethical considerations

The study was conducted after obtaining approval from the Ethics Committee of the Instituto Neumologico del Oriente (Act Number 146 CEINO-F-07), formulated according to current ethical regulations (Declaration of Helsinki/Belmont Report/CIOMS Guidelines and Resolution 8430 of 1993). Information collection was carried out after obtaining informed consent from all participants.

## Results

Results

Initial Sample Characteristics (Clinical and Sociodemographic Characteristics)

The initial sample consisted of 71 patients, of whom 47 (66.20%) had follow-up after pulmonary rehabilitation. Regarding the initial sample, the average age was 73.38 years (standard deviation -SD- 7.77); 53.52% were women; 30.99% were from Bucaramanga or its metropolitan area (Girón and Piedecuesta); 42.25% were homemakers; and 29.58% belonged to socioeconomic status 4. In terms of clinical characteristics, 60.56% were former smokers and 2.82% were still smoking at the time of measurement; 94.37% reported having some comorbidity, with hypertension (HTN) and diabetes mellitus being the most frequent, with prevalences of 56.34% and 21.13%, respectively.

Changes in Handgrip Strength

The average handgrip strength of the participants in the initial measurement was 21.47 kg (SD: 7.51), and the delta between measurements was 1.64 kg (SD: 3.48). It is worth noting that this last figure was estimated based on individuals who had data at both the initial and follow-up measurements. Other characteristics are listed in Tables 1, 2.

**Table 1:** Sociodemographic characteristics

Variable		n	%
				Female sex		38	53.52
Place of Origin		n	%
Bucaramanga and Metropolitan Area		22	30.99
Bogotá		2	2.82
Other Municipalities of Santander		39	54.93
Bolívar		1	1.41
Boyacá		2	2.82
Cesar		1	1.41
Magdalena		1	1.41
Norte de Santander		2	2.82
Quindío		1	1.41
Occupation		n	%
Unemployed		12	16.90
Driver		1	1.41
Employee		2	2.82
Home		30	42.25
Independent		6	8.45
Pensioner		20	28.17
Socioeconomic stratum		n	%
1		14	19.72
2		17	23.94
3		12	16.90
4		21	29.58
5		2	2.82
6		5	7.04

**Table 2:** Clinical Characteristics. Dyspnea and lower limb fatigue on the Borg scale were recategorized as mild (0-4) and moderate (5-10) to reduce the number of categories and increase the sample size within them. 6MWT: 6-minute walk test 1: Delta estimated in individuals with information at baseline and follow-up (n=47) 2: The median and interquartile range of 10-year survival were 21.36 (2.25-53.39) 3: The median and interquartile range of meters walked were 311 (254-395)

Variable		n	%
				Former smoker		43	60.56
Current smoker		2	2.82
Presence of any comorbidity		64	94.37
HT		40	56.34
DM		15	21.13
Dyslipidemia		11	15.49
Heart Failure		12	16.90
Coronary disease		9	12.68
Cancer		4	5.63
Other Comorbidity		50	70.42
Dyspnea mMRC scale		n	%
2		34	47.89
3		30	42.25
4		7	9.86
Initial Dyspnea (Borg Scale)		n	%
Mild (0-4)		70	98.59
Moderate (5-10)		1	1.41
Final Dyspnea (Borg Scale)		n	%
Mild (0-4)		55	77.46
Moderate (5-10)		16	22.54
Initial Lower Limb Fatigue (Borg Scale)		n	%
Mild (0-4)		70	100.00
Moderate (5-10)		0	0.00
Final Lower Limb Fatigue (Borg Scale)		n	%
Mild (0-4)		59	83.10
Moderate (5-10)		12	16.90
Variable		Media	DE
Age (Years)		73.38	7.77
Grip Strength (kg); initial measurement		21.47	7.51
Grip Strength (kg); delta between measurements^1^		1.64	3.48
Charlson Index Score		4.61	1.49
10-Year Survival ^2^		38.17	29.85
Quality of Life (St. George's Scale)		Media	DE
Symptoms		46.37	21.55
Activities		70.66	17.72
Impact		32.20	20.28
Global		46.22	17.14
6MWT		Media	DE
Meters Walked ^3^		321.97	104.14
Initial Dyspnea (Borg Scale)		0.47	0.97
Final Dyspnea (Borg Scale)		3.01	2.49
Initial Lower Limb Fatigue (Borg Scale)		0.20	0.64
Final Lower Limb Fatigue (Borg Scale)		2.20	2.23
Initial Heart Rate		80.21	13.47
Final Heart Rate		105.30	19.50
Initial Respiratory Rate		16.76	1.78
Final Respiratory Rate		35.27	4.23
Initial O2 Saturation(%)		94.04	2.01
Final O2 Saturation (%)		88.35	6.78
Spirometry		Media	DE
Forced Vital Capacity (FVC); liters		1.89	0.66
Forced Expiratory Volume (FEV); liters		1.16	0.60
FEV1/FVC Ratio (%)		60.99	15.99

Handgrip Strength Variation Between Measurements

The delta of handgrip strength (HGS) between the second and first measurements averaged 1.64 kg (SD: 3.48), with a statistically significant difference. Moreover, 74.47% (n=35) of the individuals who completed the follow-up had a positive delta, indicating higher handgrip strength in the second measurement compared to the first. Additionally, participants with a positive delta were older, with a statisticallysignificant difference compared to those with a negative delta (p = 0.003). The Charlson index was higher in participants with a positive delta compared to those with a negative delta (p = 0.01).

Regarding the delta of handgrip strength between the second and first measurements, it was found that the average change was 1.64 kg (SD: 3.48), and this variation was statistically significant (pre-handgrip strength: 21.44 kg vs. post-handgrip strength: 22.93 kg; p<0.001). Additionally, the variable was categorized based on the magnitude of change, considering a positive delta (improvement) for all differences greater than zero, while a negative delta (deterioration) was assigned when the difference was less than zero. This showed that 74.47% (n=35) of the individuals who completed follow-up had a positive delta, meaning their handgrip strength was higher in the second measurement than in the first.

The analysis of baseline clinical and spirometric characteristics according to the delta in handgrip strength revealed that individuals with a positive delta (improvement) were older and had a significantly higher Charlson index score compared to those with a negative delta.

Correlation With Quality of Life

**Figure 1:**
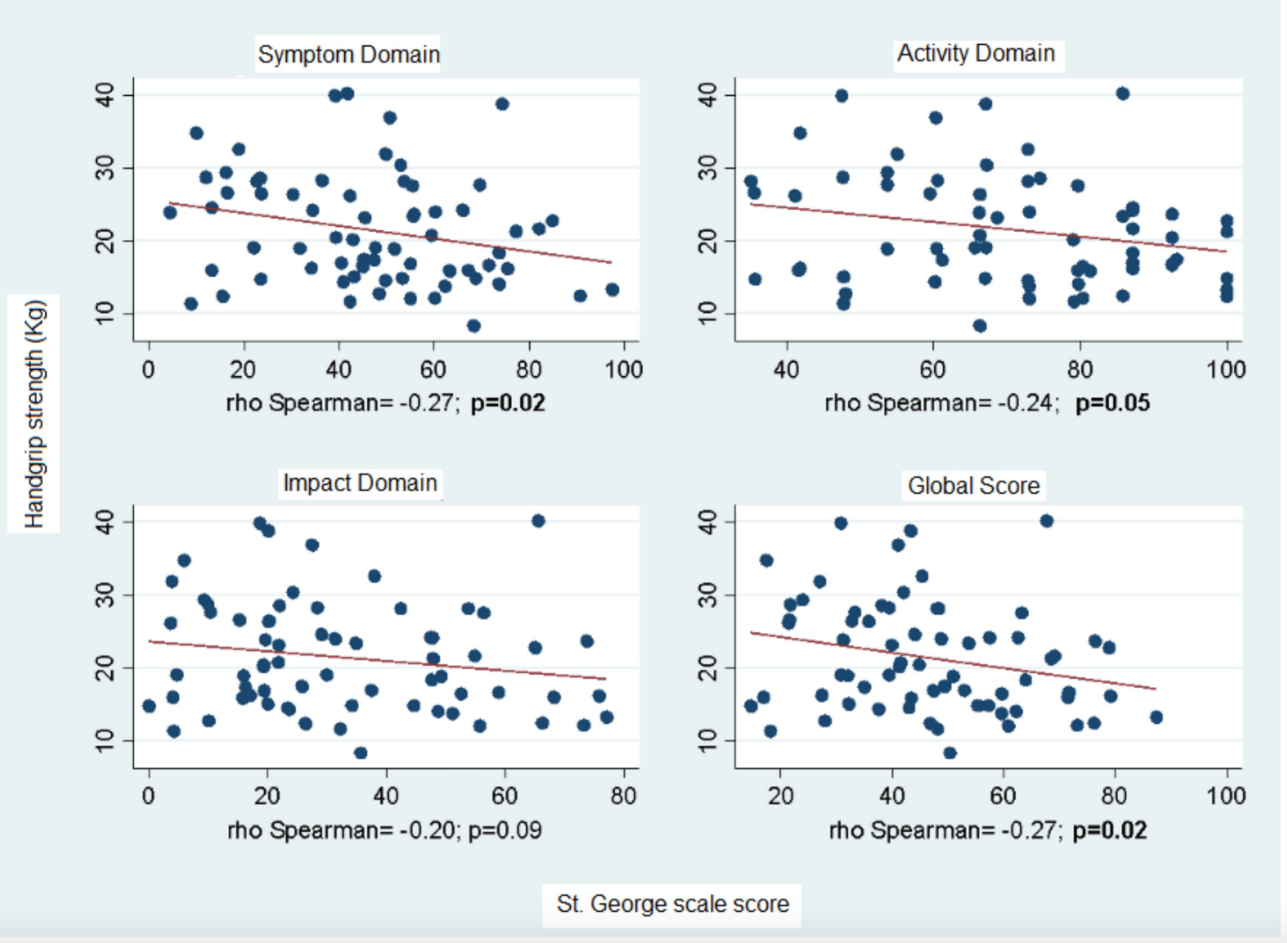
Correlation of Handgrip Strength and Quality of Life Score at Baseline Measurement.

**Figure 2:**
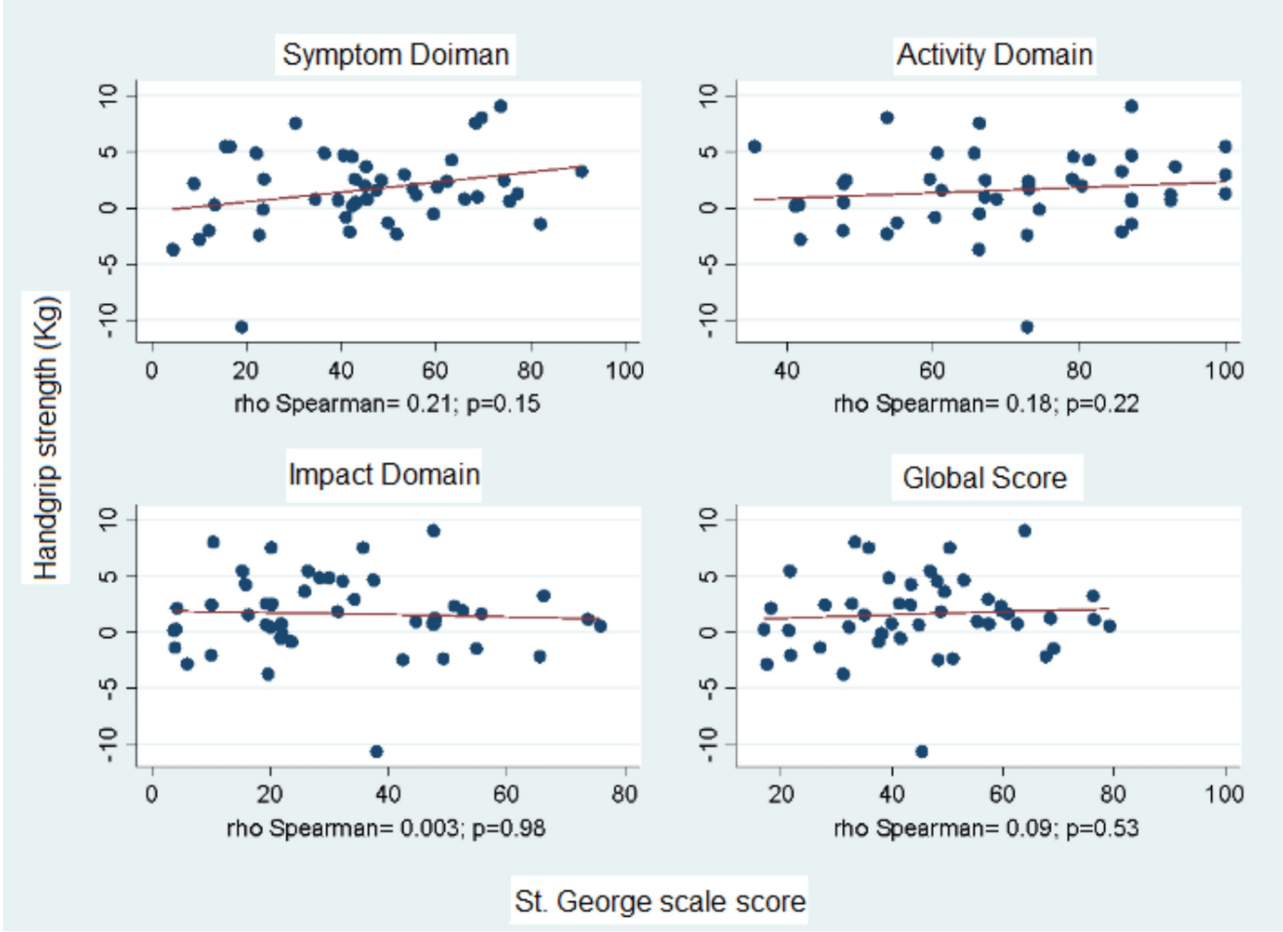
Correlation between the Delta of Handgrip Strength Pre and Post Pulmonary Rehabilitation and Initial Quality of Life.

The analysis of the relationship between quality of life, measured with the St. George's Respiratory Questionnaire (SGRQ), and baseline handgrip strength (HGS) showed a negative correlation between these two variables across all domains of the questionnaire and the overall score (see Figure 1). Conversely, the trend of correlation for the delta of handgrip strength (HGS) was positive, with higher baseline quality of life scores correlating with a positive change in handgrip strength between measurements (see Figure 2).

Correlation With Dyspnea

When evaluating the relationship between dyspnea, measured using the **mMRC** scale, and handgrip strength (HGS), a significant change was found in the average delta of handgrip strength across the levels of dyspnea (p=0.05) (see Table 3).

**Table 3:** Behavior of Handgrip Strength (Baseline and Delta) According to the Level of Dyspnea Prior to Pulmonary Rehabilitation.

Variable	Baseline Grip Strength n= 71			Delta of Grip Strength n= 47			
								Average	SD	p	Average		SD	p
	Dyspnea Scale mMRC							
2	22.70	8.21		0.63		3.95	
3	20.89	6.97	0.34	2.21		2.73	0.05
4	18.00	5.29		3.77		2.46	

Correlation With 6MWT

**Figure 3:**
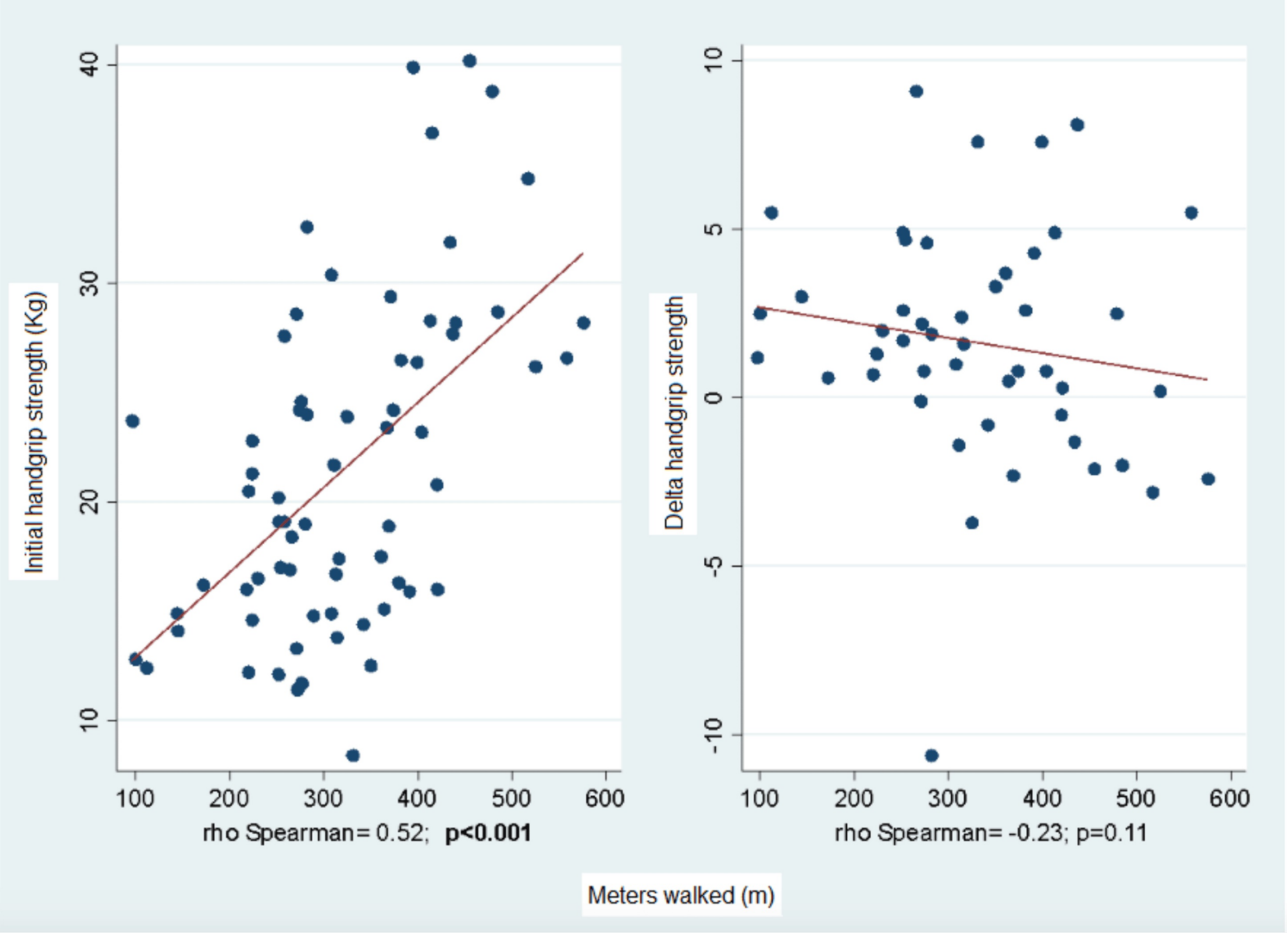
Correlation Between Initial Handgrip Strength and its Delta, and Meters Walked in the 6MWT Prior to Pulmonary Rehabilitation. 6MWT: 6-minute walk test

Lastly, a correlation was found between the parameters of the six-minute walk test (6MWT), baseline handgrip strength, and its delta, with a linear relationship between the distance walked and initial handgrip strength (see Figure 3).

## Discussion

The apparent relationship between chronic respiratory diseases and muscle strength loss has been explained from different perspectives, which may be related to decreased muscle strength, leading to functional alterations in COPD patients [3].

Previous studies have reported an average decrease of 1-2% in muscle mass and strength with aging, which is more evident in elderly individuals with COPD compared to healthy individuals, consistent with the demographic characteristics of the study population, with a mean age of 73.38 years and most having underlying comorbidities [3,8,9].

In this study, the baseline HGS of the population was 21.47 kg (SD 7.51), consistent with Fonseca J et al. (2020), where COPD patients had a dominant hand strength of 28 kg (IQR 25-38)[22]. After PR, a significant average increase of 1,6 kg in strength compared to the initial measurement was found, indicating that physical training can improve muscle strength in patients.

Patients with lower initial quality of life scores (higher percentage in the SGRQ) had lower HGS, consistent with studies by Lima et al. and Holden M et al., where these correlations were similar to our study (rho = -0.26; p 0.05) [23,24]. This may be because decreased muscle strength in individuals with chronic respiratory diseases can exacerbate disease symptoms and thus quality of life [25].

Similarly, the domain scores were consistent with those reported by Kaymaz D. et al. [26] in 2018, where all correlations (rho) were negative: -0.329 in the activity domain (p 0.002); -0.346 in the symptoms domain (p 0.001); and -0.110 in the impact domain (p 0.308). These results align with our findings, as all domains of the St. George’s questionnaire showed a negative correlation, and the impact domain also had the lowest correlation. However, in our study, the activity domain did show statistical significance (p 0.05).

Additionally, it was found that participants with a lower initial quality of life showed a greater positive delta regarding HGS and symptoms due to high metabolic load and dyspnea during daily activities involving the upper limbs. Therefore, engaging in physical training can result in a greater change in muscle strength [5,13].

Dyspnea in our population had a negative relationship with initial HGS, where strength was lower as the mMRC scale score increased.

This finding is consistent with the study by Byun MK et al., who reported that individuals with COPD with impaired HGS had higher dyspnea scores (rho -0.346 p 0.002) [27]. Similarly, it was found that, after PR, patients who had a positive change in HGS were those who initially reported a higher score on the mMRC scale.

Regarding spirometric variables, we did not find significant differences in the distribution of the behavior of FVC, FEV1, and the FEV1/FVC ratio according to the attainment of a positive or negative delta in HGS between measurements. In contrast, Martinez et al [28], in their study conducted on 272 COPD patients, found that manual strength had a positive, though small, correlation with FEV1 (rho 0.47). However, despite the decrease in muscle strength, the degree of airway obstruction remained unchanged after one year of follow-up.

The utility of HGS in COPD patients includes monitoring disease progression, assessing functional capacity, predicting clinical outcomes, detecting early signs of frailty, and guiding treatment and rehabilitation. Decreased HGS can indicate worsening disease or muscle weakness, and lower HGS is associated with poorer quality of life and higher exacerbation rates. Therefore, HGS is a valuable tool for evaluating and managing COPD patients.

The study recognizes limitations such as a higher than estimated loss to follow-up rate (approximately 30%), which could induce selection bias and reduce statistical power. Nevertheless, a sensitivity analysis of the baseline characteristics of individuals with and without follow-up showed no statistically significant differences in the analyzed variables. Furthermore, there could have been information bias due to differential recall according to the severity of the patient's condition; however, this was mitigated through the application of the instrument by previously trained personnel. Another confounding factor that could have influenced the results is the lack of consideration of the pharmacological treatment the patients were receiving and, in the case of women, their menopausal status. The literature suggests that menopause in women can decrease handgrip strength [29] Additionally, some pharmacological therapies are more effective than others, which could impact the improvement of handgrip strength in patients [30]. However, it is important to emphasize that the primary objective of this study was to evaluate handgrip strength. It should also be noted that, due to the SARS-CoV-2 pandemic, it was challenging to obtain a representative sample of patients attending rehabilitation therapy in person.

Given these limitations, future studies should consider including more detailed demographic and clinical variables, such as pharmacological treatments and menopausal status, to better understand their impact on muscle strength in COPD patients. Additionally, larger sample sizes should be sought to enhance the generalizability of the findings.

## Conclusions

This study highlights the importance of periodic handgrip strength measurements to predict frailty and muscle dysfunction in COPD patients, as well as the positive effect of pulmonary rehabilitation therapy as a simple and cost-effective intervention that correlates with the improvement of indirect prognosis and survival indicators, which could potentially influence disease control and, consequently, reduce disease burden and improve patients' quality of life.

However, further research is recommended to more precisely define the impact of pulmonary rehabilitation on muscle strength and its relationship with other clinical factors in COPD patients.
